# Applications for the 2019 ECDC traineeship programme, including at the *Eurosurveillance* office, open until 31 January

**DOI:** 10.2807/1560-7917.ES.2019.24.4.1901241

**Published:** 2019-01-24

**Authors:** Eurosurveillance editorial team

**Affiliations:** 1 European Centre for Disease Prevention and Control (ECDC), Stockholm, Sweden

The European Centre for Disease Prevention and Control (ECDC) invites applications for the 2019 ECDC traineeship programme including the following areas.

***Eurosurveillance* editorial office:** the office is responsible for *Eurosurveillance*; Europe's journal on infectious disease surveillance, epidemiology, prevention and control published by ECDC.

**Vaccine-preventable diseases:** the programme is responsible for epidemic intelligence, health communication, microbiology, preparedness and response, public health training, scientific advice and surveillance for vaccine-preventable diseases.

**Influenza and other respiratory viruses**: the programme is responsible for epidemic intelligence, health communication, microbiology, preparedness and response, public health training, scientific advice and surveillance for influenza and other respiratory viruses.

**Stakeholder engagement:** the function is responsible for improving the communication with ECDC's stakeholders. The tasks of the trainee will be shared between the communication and the public health training section.

**Country capacity support and capacity gap analysis**: the section is responsible for coordinating ECDC's activities in planning and evaluation of emergency preparedness and other systems for communicable disease control.

For more information, please visit the ECDC website: https://ecdc.europa.eu/en/about-uswork-us/ecdc-traineeship-programme

